# Notch Signaling Molecules Activate TGF-**β** in Rat Mesangial Cells under High Glucose Conditions

**DOI:** 10.1155/2013/979702

**Published:** 2013-04-18

**Authors:** Li Liu, Chenlin Gao, Guo Chen, Xia Li, Jia Li, Qin Wan, Yong Xu

**Affiliations:** ^1^Department of Endocrinology, The Affiliated Hospital of Luzhou Medical College, Luzhou, Sichuan, China; ^2^Department of Endocrinology, The People's Hospital of Yongchuan, Yongchuan, Chongqing, China; ^3^Department of Endocrinology, The First Hospital of Yibin, Yibin, Sichuan, China

## Abstract

The involvement of the Notch signaling pathway in the cellular differentiation of the mammalian kidney is established. Recently, the dysregulation of Notch signaling molecules has been identified in acute and chronic renal injuries, fibrosis models, and diabetic kidney biopsies. The canonical Notch ligand , Jagged1, is upregulated in a transforming growth factor-beta- (TGF-**β**-) dependent manner during chronic kidney disease. TGF-**β**, a central mediator of renal fibrosis, also is a major contributor to the development of diabetic nephropathy. To explore the roles and possible mechanisms of Notch signaling molecules in the pathogenesis of diabetic nephropathy, we exposed cultured rat mesangial cells to a **γ**-secretase inhibitor (DAPT) or high glucose and measured the expression of Notch signaling molecules and the fibrosis index. Notch pathway-related molecules, TGF-**β**, and fibronectin increased with exposure to high glucose and decreased with DAPT treatment. Our results suggest that the Notch signaling pathway may precipitate diabetic nephropathy via TGF-**β** activation.

## 1. Introduction

Diabetic nephropathy is a common microvascular complication of diabetes with a poorly understood pathogenesis. Hemodynamic changes and disorders of glucose metabolism resulting from genetic factors, hyperglycemia, and/or the actions of angiotensin II and other cytokines can precipitate in the development of diabetic nephropathy. Notably, activation of the Notch signaling pathway can induce the formation of glomerular and tubular lesions that are characteristic of this disease [1, 2]. The Notch ligand, Jagged1, and its target gene product, Hes1, are elevated in renal biopsies from diabetic nephropathy patients, further implicating Notch activation in this disease [3–5]. Despite evidence that Notch activation is present in diabetic nephropathy specimens *in vivo*, no reports have investigated Notch signaling during the development of diabetic nephropathy.

The early stages of diabetic nephropathy are associated with changes in certain cytokines, growth factors, and adhesion molecules, including TGF-*β*, a central mediator of the fibrotic response. Enhanced fibronectin (FN) deposition ultimately leads to glomerulosclerosis and tubulointerstitial fibrosis, which are characteristics of end-stage diabetic nephropathy [6]. The levels of Jagged1, Jagged2, and Notch1, 4 were upregulated significantly in a human kidney epithelial cell line (CC-2554) with TGF-*β*1 treatment in a dose-dependent manner [7]. Whether the Notch signaling pathway is involved directly in the pathogenesis of diabetic nephropathy remains unclear.

To elucidate the molecular events involved in Notch signaling that may precipitate diabetic nephropathy, we investigated whether abnormal levels of Notch could initiate signs of diabetic nephropathy in an *in vitro *model. To determine the effects of high glucose on Notch pathway molecules, glomerular mesangial cells (GMCs) were cultured with various concentrations of glucose (5.6, 15, 25, or 35 mmol/L) and different times (12 h, 24 h, 48 h). The expression levels of Notch pathway components in GMC then were measured using Western blotting, RT-PCR, and immunofluorescent staining and laser scanning confocal microscopy. Our preliminary results support that Notch signaling molecules may contribute to this disease via TGF-*β* activation.

## 2. Materials and Methods

### 2.1. Cell Culture

Rat mesangial cells (HBZY-1) were cultured at 37°C with 5% CO_2_ in low glucose DMEM containing 10% fetal calf serum and 1% penicillin/streptomycin. Cells were analyzed at the first and third passages. Once cells reached 90% confluency, they were serum deprived in 0.2% FBS for 24 h. Subsequently, cells were stimulated with various media in the absence or presence of glucose at various concentrations. Prior to high-glucose exposure, some cells were exposed to 1 *μ*mol/l N-(N-(3, 5-difluorophenacetyl)-l-alanyl-S-phenyl-glycine t-butylester (DAPT), which can inhibit Notch pathway that was added to the low glucose medium for 2 h. GMCs were randomly divided into the following six groups:

- the normal control group (NC group), with medium containing 5.6 mmol/L glucose;
- the 15 mmol/L glucose group (HG1 group), with medium containing 15 mmol/L glucose;
- the 25 mmol/L glucose group (HG2 group), with medium containing 25 mmol/L glucose;
- the 35 mmol/L glucose group (HG3 group), with medium containing 35 mmol/L glucose;
- the osmotic pressure group as a control (OP group), with medium containing 5.6 mmol/L glucose +19.4 mmol/L mannitol;
- the DAPT intervention group (HD group), with medium containing 25 mmol/L glucose +1 *μ*mol/L DAPT.

### 2.2. RNA Isolation and Reverse Transcription-PCR

Cells were collected at 12 h, 24 h, or 48 h time points following glucose or DAPT exposure and immediately were washed in ice-cold phosphate-buffered saline (PBS). Cells then were lysed in 1 mL of TRIzol reagent, and total RNA was isolated as described previously. Samples were reverse transcribed (RT) to cDNAs using SuperScript II reverse transcriptase (Chengdu, Break) according to the manufacturer's instructions. The cDNAs were amplified in a 25 *μ*L PCR mixture containing template cDNA (4 *μ*L), 1 *μ*L each forward and reverse primers, and 12.5 *μ*L 2× FTC2000 PCR Master Mix (Funglyn Biotech, Canada). Primers were targeted to amplify Jagged1, Notch1, Hes1, FN, TGF-*β*, and GAPDH gene regions (Shengong, Shanghai, China). The following primer sequences were used: Notch1, sense, 5′-CAT CTC CGA CTT CAT CTA TC-3′, antisense, 5′-TCT CCT CCT TGT TGT TC TG-3′; Jagged1, sense, 5′-GCT GGG AAG GAA CAA CC-3′, antisense, 5′-CCT GGA GGG CAG ATA CAC-3′; Hes1, sense, 5′-CGG ACA AAC CAA AGA CC-3′, antisense, 5′-AAG CGGGTC ACC TCG TTC A-3′; FN, sense, 5′-TGC CGA ATG TAG ATG AGG A-3′, antisense, 5′-AAA TGA CC ACT GCC AAA GC-3′; TGF-*β*, sense, 5′-ATG GTG GAC CGC AAC AAC-3′, antisense, 5′-GAG CAC TGA AGC GAA AGC-3′; GAPDH, sense, 5′-CCT CAA GAT TGT CAG CAA T-3′, antisense, 5′-CCA TCC ACA GTC TTC TGA GT-3′.

### 2.3. Western Blotting

Harvested cells were washed gently with PBS twice and then were pelleted at 1000 rpm for 5 min at 4°C. Cell pellets were lysed in 300 *μ*L of sucrose buffer (0.125 mL 1.0 M Tris pH 7.5, 0.125 mL 3.0 M NaCl, 25 *μ*L 10% SDS, 25 *μ*L TritonX, 100 *μ*L protease inhibitor) per 100 mm culture dish. Lysates were shaken on ice for 15 minutes, and whole-cell protein extracts were obtained. SDS-PAGE sample loading buffer (5×) was added, and cell fractions were boiled in 95°C asepsis water. Proteins were probed with antibodies raised against Jagged1 (rabbit, 1 : 400, YiXin Company, China), Notch1 (Zhongshan zs-6014, Beijing, China), Hes1 (rat, 1 : 400, Santa Cruz, USA), and *β*-actin (mouse, 1 : 1000, Beyotime Institute of Biotechnology, China). TGF-*β* antibody (rabbit, 1 : 1000, CST, USA), GAPDH (mouse, 1 : 1000, Beyotime, China), and Smad4 antibody (rabbit, 1 : 500, Santa Cruz, USA).

### 2.4. Immunofluorescence Microscopy

We treated GMC with 5.6 mmol/L or 25 mmol/L glucose in the presence or absence of DAPT for 24 h. Cells were grown on coverslips in 6-well plates. After overnight adherence, the cells were treated with media containing high glucose, mannitol, and the DAPT media for 24 h. The cells were fixed in 4% paraformaldehyde (Pierce) and permeabilized in 0.2% Tween 20 (Sigma) for 10 min after being washed briefly with PBS. The cells were blocked with 5% serum for 1 h at room temperature and incubated overnight with primary antibodies followed by washes with PBS. The cells were incubated for 40 min with the appropriate secondary antibody conjugated to the FITC fluorescent dye. The coverslips were washed and mounted onto slides using fluorescent mounting medium (Beyotime, Shanghai, China). The control cells were incubated without a primary antibody. Images were taken with a DMIRE_2_ laser scanning confocal microscope (Leica, Germany).

The following antibodies were used for immunofluorescence: goat anti-Notch1 (1 : 50; zs-6014; Beijing, China), rat anti-Hes1 (1 : 50; sc-166378), and rabbit anti-Jagged1 (1 : 50 Yixin, Shanghai, China).

### 2.5. Statistical Analyses

All values are represented as means ± standard errors (S.E.) from at least three independent experiments. Statistical significance was assessed using ANOVA. Significance was set at *P* < 0.05. All data were analyzed using SPSS statistical software.

## 3. Results

**Figure 1:**
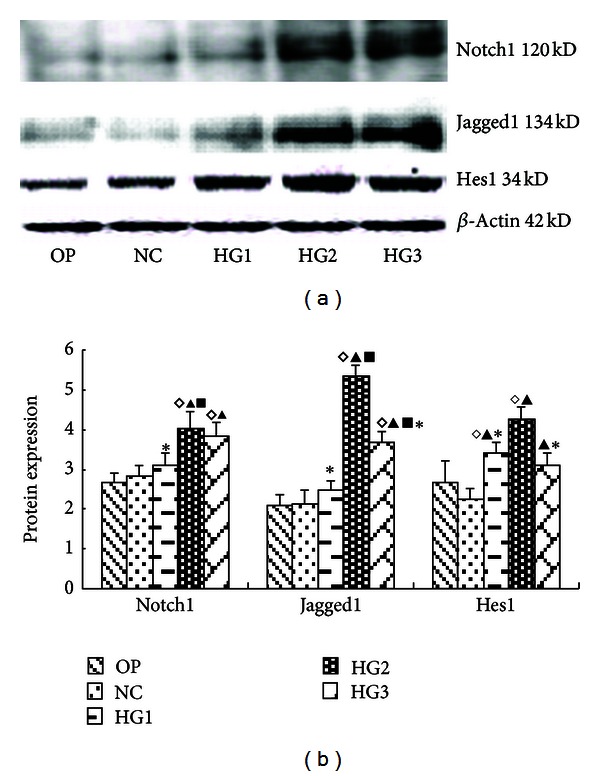
The expression of Notch pathway components as measured by Western blotting following treatment of GMC with various glucose concentrations for 24 h. (a) Notch-associated molecule proteins at different glucose concentrations and high osmotic pressure at 24 h. Notch-associated molecules increased in the high glucose group; they had the most significant changes in the HG2 group, but there were no apparent differences between the NC group and the OP group. (b) The gray graph shows the relative statistical values of Notch-associated molecules for each group. The expression of the Notch-associated molecules increased in the high glucose group, especially, in the HG2 group. ^*◊*^ *P* < 0.05 versus OP group; ^▴^ *P* < 0.05 versus NC group, ^▪^ *P* < 0.05 versus HG group; *P < 0.05 versus HG2 group.

**Figure 2:**
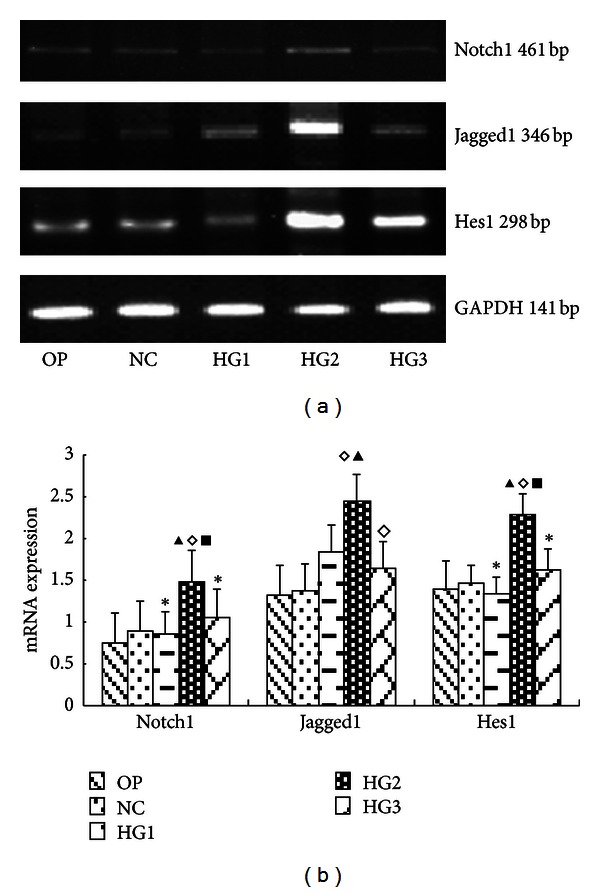
The mRNA levels of Notch pathway components in each group of GMCs. (a) RT-PCR strip chart for different concentrations of glucose. pathway components mRNA increased in the high glucose group, especially in HG2 group. (b) The corresponding relative gray value statistics graph of the mRNA level. ^*◊*^ *P* < 0.05 versus OP group; ^▴^ *P* < 0.05 versus NC group; ^▪^ *P* < 0.05 versus HG1 group; *P < 0.05 versus HG2 group.

After 24 h culture, compared with normal glucose controls, the protein expression levels of all Notch signaling molecules were significantly increased in GMC in HG2 group (*P* < 0.05). In the OP group, the expression of Notch pathway components was statistically similar to NC Group (*P* > 0.05) (Figure 1). RT-PCR confirmed these trends with respect to mRNA expression (Figure 2).

**Figure 3:**
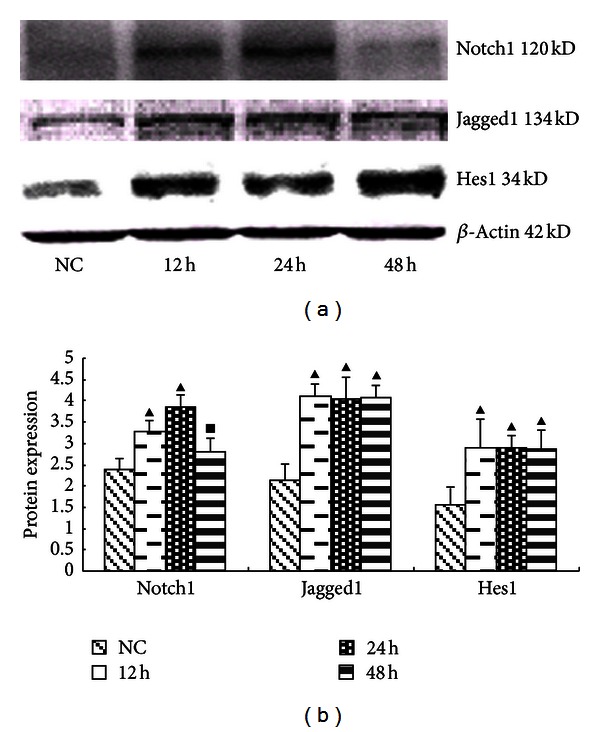
The expression of Notch pathway components in GMCs induced by 25 mmol/L glucose at various times determined using Western blot. (a) Western Blot strip chart for different times. At the 24 h time point, all Notch-related proteins were significantly enhanced. Notch1 protein was increased at both the 12 h and 24 h time points, with the 24 h increase being more pronounced. (b) The gray value statistics graph of the protein level. ^▴^ *P* < 0.05 versus NC group; ^•^ *P* < 0.05 versus 12 h group; ^▪^ *P* < 0.05 versus 24 h group.

**Figure 4:**
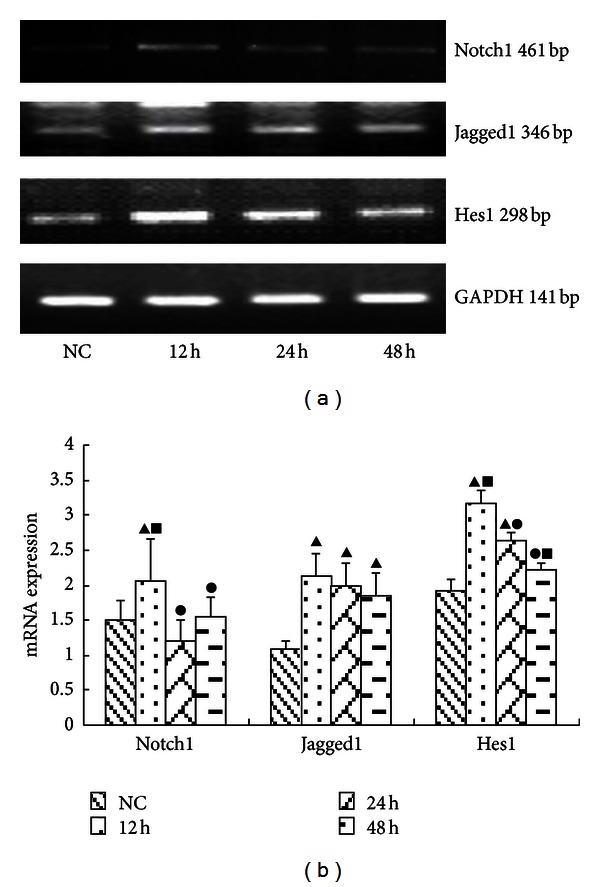
The mRNA levels of FN in each group of GMCs. (a) RT-PCR strip chart for different times. The mRNA expression of all Notch signaling components were significantly upregulated at the 12 h time point. The expression of *Notch1* mRNA was enhanced significantly at the 12 h time point. The expression levels of *Hes1* mRNA were upregulated significantly at the 12 h and 24 h (*P* < 0.05) time points, with the 12 h increase being more obvious. (b) The corresponding relative gray value statistics graph of the mRNA level. ^▴^ *P* < 0.05 versus NC group; ^•^ *P* < 0.05 versus 12 h group; ^▪^ *P* < 0.05 versus 24 h group.

Cells cultured in medium with or without high glucose (25 mmol/L) were harvested at various time points following exposure (12 h, 24 h, or 48 h). At the 24 h time point, all Notch-related proteins were significantly enhanced (*P* < 0.05). Notch1 protein was increased at both the 12 h and 24 h time points, with the 24 hr increase being more pronounced (Figure 3). The mRNA expression levels of all analyzed Notch signaling components were significantly upregulated at the 12 h time point (*P* < 0.05). The expression of Notch1 mRNA was enhanced significantly only at the 12 h time point. The expression levels of Hes1 mRNA were upregulated significantly at the 12 h and 24 h (*P* < 0.05) time points, with the 12 h increase being more obvious (Figure 4).

**Figure 5:**
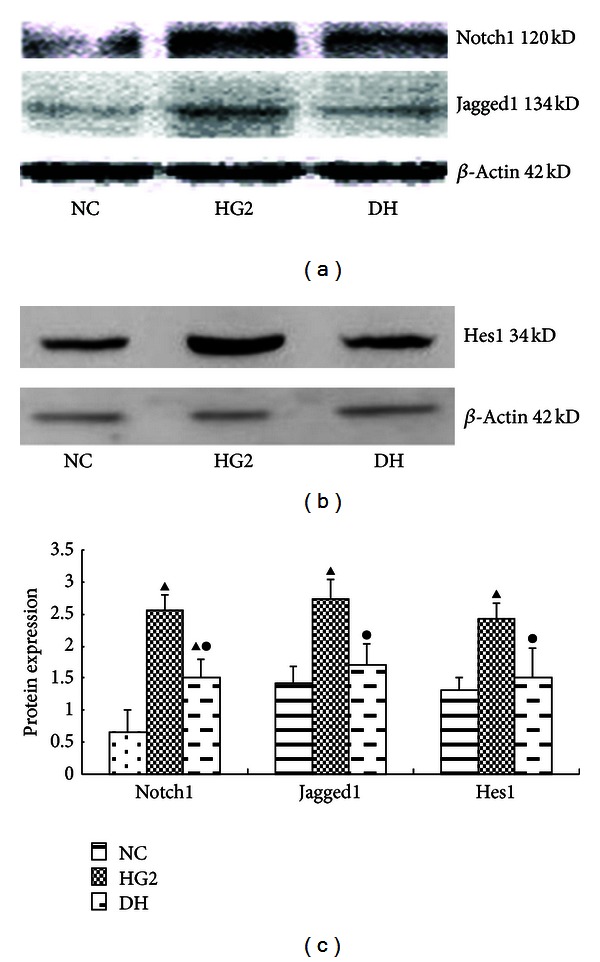
The expression of Notch pathway components after intervention with DAPT determined using western blot for 24 h. (a) Western blot strip chart for Notch1 and Jagged1. (b) Western blot strip chart for Hes1. (c) The gray graph shows the relative statistical values for Notch pathway components in each group. Compared with the NC group, the expression of the Notch pathway components protein was significantly increased in the HG2 group. After DAPT intervention, the protein expression of Notch pathway components decreased. ^▴^ *P* < 0.05 versus NC group; ^•^ *P* < 0.05 versus HG2 group.

**Figure 6:**
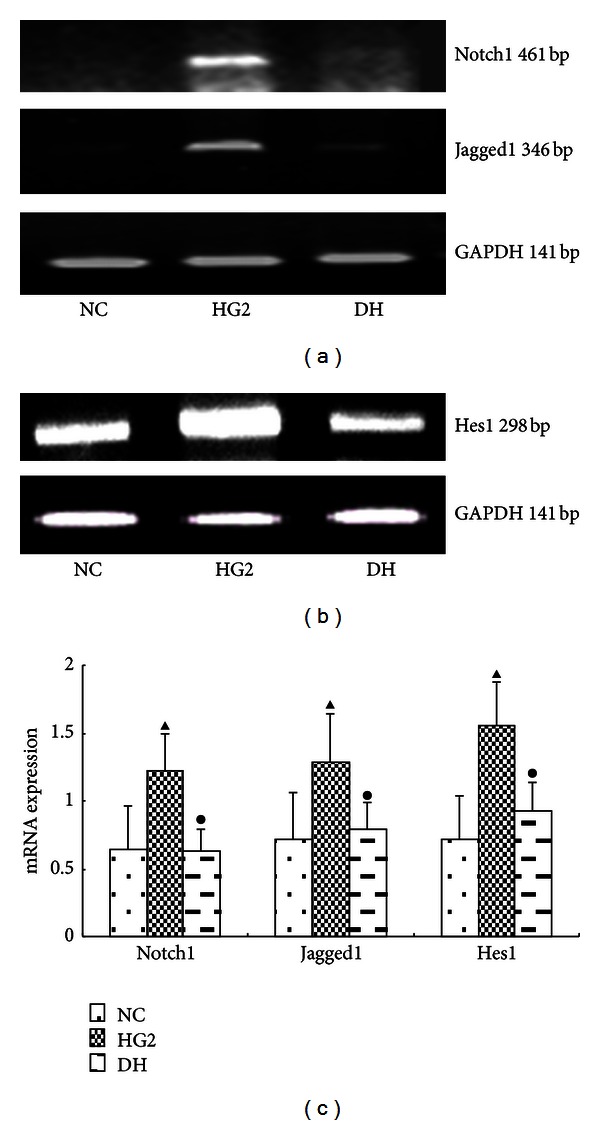
The expression of Notch pathway components after intervention with DAPT determined using PCR for 12 h. (a) RT-PCR strip chart for Notch1 and Jagged1. (b) RT-PCR strip chart for Hes1. (c) The gray graph shows the relative statistical values for Notch pathway components in each group. Compared with the NC group, the expression of the Notch pathway components mRNA was significantly increased in the HG2 group. After DAPT intervention, the mRNA expression of Notch pathway components decreased. ^▴^ *P* < 0.05 versus NC group; ^•^ *P* < 0.05 versus HG2 group.

Following treatment with DAPT, the protein expression levels of all Notch pathway components (Notch1, Jagged1, and Hes1) were decreased significantly (*P* < 0.05) (Figure 5). The same results were detected using RT-PCR (Figure 6).

**Figure 7:**
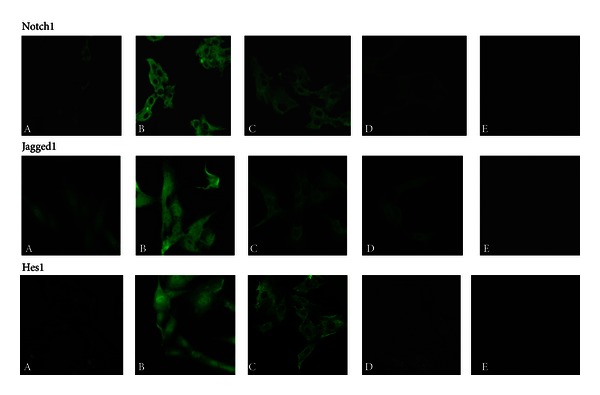
The expression of Notch pathway components in GMCs by immunofluorescent staining and laser scanning confocal microscopy applications. A, NC group; B, HG2 group; C, HD group; D, OP group; E, Negative control. Notch pathway component proteins were detected in the cell as green fluorescence. The Notch pathway components protein was little detected in the NC group, but it was prominent in the HG2 group, which did not significantly change as the result of high osmotic pressure. The protein was less detected in the DAPT intervention group. Original magnification ×630.

In the cell immunofluorescent staining and laser scanning confocal microscopy applications, the Notch1 protein was weakly expressed in the cytoplasm of cells cultured in 5.6 mmol/L glucose. Following treatment with 25 mmol/L high glucose for 24 h, Notch1 expression levels increased significantly in the cytoplasm but remained undetectable in GMC nuclei. In GMC treated with DAPT, the expression of Notch1 was decreased significantly in the cytoplasm compared with cells exposed to 25 mmol/L glucose group. The Jagged1 protein was expressed in the cytoplasm and nuclei of cells cultured under normal glucose conditions. Upon treatment with 25 mmol/L glucose for 24 h, Jagged1 expression increased significantly. Compared with the 25 mmol/L glucose group, the expression of Jagged1 in the DAPT-exposed group was decreased in cytoplasmic and nuclear compartments, with weaker expression in cell nuclei. The Hes1 protein was weakly expressed in the cytoplasm of GMC treated with 5.6 mmol/L glucose. Following treatment with 25 mmol/L glucose for 24 h, Hes1 expression increased significantly in the cytoplasm and nuclei of GMC. The levels of Hes1 protein in the DAPT-exposed group were decreased in cytoplasmic and nuclear compartments, compared with the high glucose-treated group, with weaker expression in nuclei than in the cytoplasm (Figure 7).

**Figure 8:**
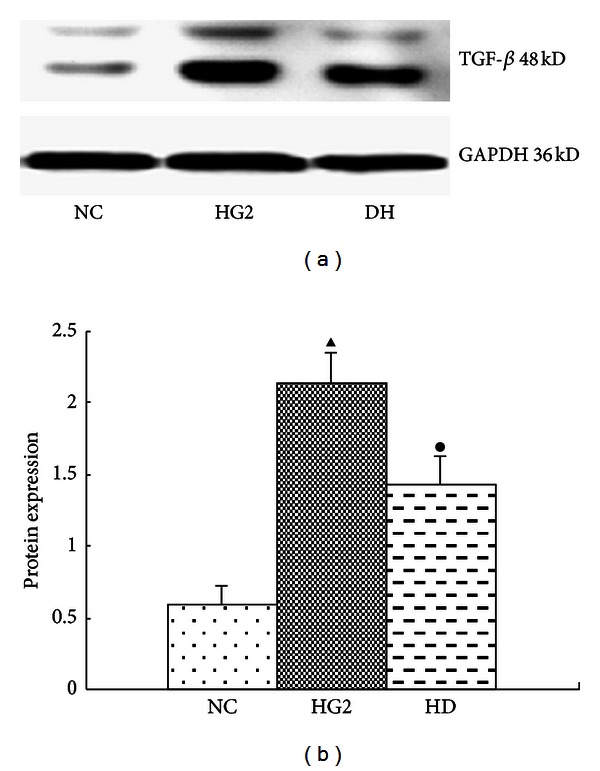
The expression of TGF-*β* after intervention with DAPT determined using western blot for 24 hr. (a) Western blot strip chart. (b) The gray graph shows the relative statistical values for TGF-*β* in each group. Compared with the NC group, the expression of the TGF-*β* protein was significantly increased in the HG2 group. After DAPT intervention, the protein expression of TGF-*β* decreased. ^▴^ *P* < 0.05 versus NC group; ^•^ *P* < 0.05 versus HG2 group.

**Figure 9:**
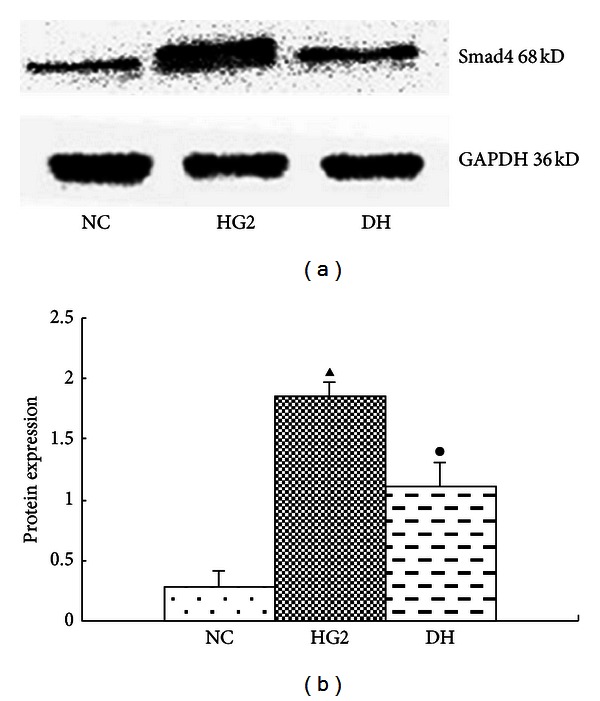
The expression of Smad4 after intervention with DAPT determined using western blot for 24 hr. (a) Western blot strip chart. (b) The gray graph shows the relative statistical values for Smad4 in each group. Compared with the NC group, the expression of the Smad4 protein was significantly increased in the HG2 group. After DAPT intervention, the protein expression of Smad4 decreased. ^▴^ *P* < 0.05 versus NC group; ^•^ *P* < 0.05 versus HG2 group.

**Figure 10:**
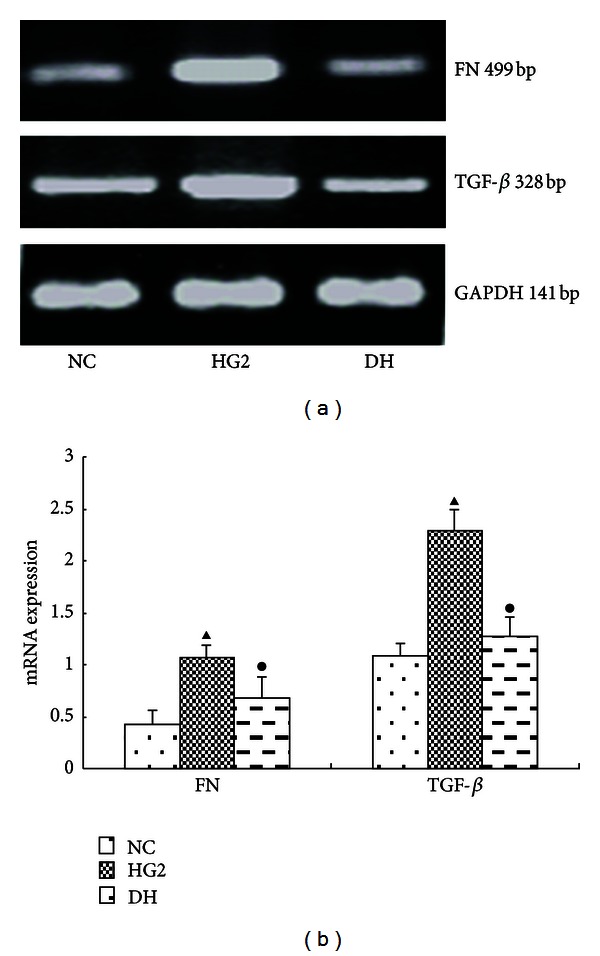
The expression of FN and TFG-*β* after intervention with DAPT determined using PCR for 12 hr. (a) RT-PCR strip chart for FN and TFG-*β*. (b) The gray graph shows the relative statistical values for FN and TFG-*β* in each group. Compared with the NC group, the expression of the FN and TFG-*β* mRNA was significantly increased in the HG2 group. After DAPT intervention, the mRNA expression of FN and TFG-*β* decreased. ^▴^ *P* <0.05 versus NC group; ^•^ *P* < 0.05 versus HG2 group.

Compared with the NC group, the expression of the TGF-*β* and Smad4 protein was significantly increased in the HG2 group. After DAPT intervention, they decreased (Figures 8 and 9). The mRNA expression of the FN and TFG-*β* changed in the same trend (Figure 10).

## 4. Discussion

Notch proteins are a family of single transmembrane proteins. In mammals, four Notch genes specify four types of Notch receptors (Notch1, 2, 3, and 4) [8, 9]. All Notch-associated proteins contribute to processes involved in kidney development. Hes1 is a Notch target gene that encodes a transcriptional regulator. The expression status of Hes1 is a marker for activation of the Notch signaling pathway [10]. Notch1 may participate in the development of fibrosis, which is correlated with glomerular sclerosis [11].

Aberrations in the expression patterns of Notch pathway components affect normal development of the kidney [12]. By assessing the expression of Notch-associated molecules, one can indirectly measure the strength of Notch signaling. Moreover, the expression, transport, and degradation of Notch-related molecules are subject to several regulatory factors [13]. For instance, the interaction of the Notch receptor with its ligand near the cell surface can trigger the Notch signaling pathway and subject the receptor to second and third digestion reactions [14]. After the third digestion reaction, Notch can be activated, and the Notch intracellular domain (NICD) can be released into the cytoplasm for subsequent translocation to the nucleus. In the nucleus, the NICD combines with the DNA binding protein CSL to activate the transcription of target genes (Hes, Hey, and Snail) with the Mam cofactor and p300 coactivator [15–17]. In addition to regulation at the receptor-ligand level, the Notch pathway is modulated by ubiquitination, degradation, protein transport levels, and so on.

A previous report suggested that the expression levels of Jagged1, Notch1, and Hes1 were increased in cultured podocytes and human embryonic kidney cells following treatment with high glucose [18]. Similarly, we found that high glucose can activate Notch signaling (i.e., increase Notch1 expression) and upregulate related molecules in mesangial cells. Jagged1 and TGF-*β* expression correlate with glomerular lesions in a diabetic rat model and with diabetic focal segmental glomerulosclerosis in patients. Inactivation of genes related to the Notch signaling pathway can reduce fibrosis in tubular epithelial cells and increase the specificity of Notch1, causing excessive proteinuria [19] and accelerating the development of tubulointerstitial fibrosis [20]. Jagged1 is upregulated in a ureteral obstruction model in a TGF-*β*-dependent manner [7].

The TGF-*β* signaling pathway is composed of the TGF-*β* superfamily, the TGF-*β* receptor, the Smads protein family, and its regulatory gene. Each component represents subtypes [21]. The TGF-*β* superfamily includes the TGF-*β* subfamily (TGF-*β*1–6), activin, and bone morphogenetic proteins [22]. TGF-*β* can increase the expression of Jagged1 and Hes1 in human kidney (HK) epithelial cells [5]. Following treatment with TGF-*β*1 for 24 h, Jagged1 and Hes1 mRNAs are increased in HK-2 cells [23]. In our study, we find that the protein expression of Smad4 increased significantly in the HG2 group. After DAPT intervention, the protein expression of Smad4 decreased. This revealed that TGF-*β* pathway indeed is activated in our study.

TGF-*β*1 can promote the synthesis and deposition of the mesangial matrix, and TGF-*β* is a key factor in the development of renal fibrosis [24, 25] and tubulointerstitial fibrosis [26]. Previous studies have identified a positive feedback loop between TGF-*β* and Notch signaling in keratinocytes, and the upregulation of TGF-*β* can increase the expression of the Notch ligand, Jagged1 [27, 28]. Our study shows that the expression levels of Notch signaling molecules were significantly decreased in all DAPT-exposed groups compared with cells treated with high glucose in the absence of DAPT. This finding indicates that DAPT can inactivate the Notch pathway despite a high glucose background.

Although our experiments indicate that high glucose exposure can activate Notch signaling and that DAPT can reduce the expression of TGF-*β* and FN, there is no convincing evidence to prove that DAPT can prevent the occurrence of diabetic nephropathy. Thus, additional studies using animal models are warranted to confirm our *in vitro* results.

## 5. Summary

We report that high glucose can upregulate the expression of Notch signaling in GMC while also upregulate the expression of TGF-*β*, Smad, and FN. Activation of the Notch signaling pathway could induce TGF-*β* signaling pathway, which is involved in the pathogenesis of diabetic nephropathy.
